# Bipolar Androgen Therapy Followed by Androgen Receptor Inhibition as Sequential Therapy for Prostate Cancer

**DOI:** 10.1093/oncolo/oyad055

**Published:** 2023-04-07

**Authors:** Samuel R Denmeade, Laura A Sena, Hao Wang, Emmanuel S Antonarakis, Mark C Markowski

**Affiliations:** The Johns Hopkins Kimmel Comprehensive Cancer Center, Baltimore, MD, USA; The Johns Hopkins Kimmel Comprehensive Cancer Center, Baltimore, MD, USA; The Johns Hopkins Kimmel Comprehensive Cancer Center, Baltimore, MD, USA; University of Minnesota Masonic Cancer Center, Minneapolis, MN, USA; The Johns Hopkins Kimmel Comprehensive Cancer Center, Baltimore, MD, USA

**Keywords:** prostate cancer, castration-resistant, bipolar androgen therapy, androgen receptor

## Abstract

Inhibition of androgen receptor (AR) signaling has been the mainstay of treatment of advanced prostate cancer (PCa) for the past 80 years. Combination and sequential AR-inhibiting therapies are highly effective palliative therapy, but they are not curative. All patients eventually develop resistance to primary castrating therapy [ie, castration-resistant PCa (CRPC)]. At this point, they are treated with subsequent lines of secondary AR inhibitory therapies. However, resistance to these agents also develops and patients progress to a state we have termed complete androgen inhibition-resistant PCa. This phase of the disease is associated with poor prognosis. At this point, treatment shifts to non-hormonal cytotoxic therapies (eg, chemotherapy and radiopharmaceuticals). However, the majority of PCas remain addicted to signaling through AR throughout the course of the disease. Resistant PCa cells adaptively upregulate AR activity, despite castration and AR inhibitors, via mechanisms such as AR overexpression, gene amplification, mutation, and expression of ligand-independent variants to permit sustained liganded and non-liganded AR signaling. Studies dating back nearly 30 years indicate that high expression of AR induced by prolonged castration becomes a vulnerability of CRPC cells in vitro and in mouse xenografts to supraphysiologic androgen (SPA), which induces cell death and growth arrest in this context. Based on these studies, we developed a counterintuitive treatment called bipolar androgen therapy (BAT) for patients with CRPC, in which SPA is administered intermittently to result in cycling of serum testosterone from the polar extremes of supraphysiologic to near-castrate levels. This rapid cycling is intended to disrupt the adaptive of AR regulation associated with chronic exposure to high or low levels of testosterone, while simultaneously targeting the spectrum of AR expression present in heterogeneous CRPC tumors. We have now tested BAT in >250 patients with CRPC. Here we present a review of these clinical studies, which have demonstrated collectively that BAT can be safely given to men with CRPC, improves quality of life, and produces therapeutic responses in ~30% of patients. As expected, resistance to BAT is associated with adaptive downregulation of AR expression. Intriguingly, this downregulation is associated with restoration of sensitivity to subsequent AR inhibitor therapies.

## Teaser

The authors present a review of their clinical studies of bipolar androgen therapy for patients with castration-resistant prostate cancer, in which supraphysiologic androgen is administered intermittently to result in cycling of serum testosterone from the polar extremes of supraphysiologic to near-castrate levels.

**Implications for Practice**

Bipolar androgen therapy (BAT) is a new treatment concept for men whose prostate cancer is resistant to standard hormone-blocking therapy. Here the authors review results of clinical studies that were performed to test BAT in asymptomatic men with castration-resistant prostate cancer. The key findings are that BAT is safe in asymptomatic men with metastatic castrate-resistant prostate cancer, does not cause prostate cancer progression, shows clinical response with decreased PSA and tumor regression in 30%–40% of patients, and can reverse resistance and prolong response to subsequent antiandrogen therapy. These results support further testing of BAT as a new kind of prostate cancer therapy.

## Introduction

### The Current Treatment Paradigm

In 1941, Dr Charles Huggins reported on the remarkable palliative benefit of surgical and medical castration in men with symptomatic, metastatic prostate cancer (PCa). Since that discovery, inhibition of androgen receptor (AR) function through androgen deprivation (ADT) and direct AR inhibition has remained the mainstay of treatment.^1,2^ The current treatment paradigm for metastatic PCa is to treat men with ADT in combination with AR signaling inhibitors (ARSI) or chemotherapy until disease progression. From the very outset of ADT use, it was recognized that all men eventually develop resistance to primary ADT, a disease stage known as castration resistant PCa (CRPC). This resistance was presumed to be due to sustained AR signaling via non-prostate sources of androgens.^2^ Once the patient develops CRPC, additional AR inhibiting therapies are administered with decreasing effectiveness as more lines of therapy are given.^3,4^ In addition to therapeutic resistance, chronic exposure to ADT leads to excess morbidity and, in a subset of men, may lead to transdifferentiation to a more aggressive and lethal neuroendocrine phenotype.^5^

A number of ARSIs have been developed as first-line therapy in combination with or after ADT aimed at further blocking androgen signaling through AR.^6^ These include the CYP17 androgen-synthesis inhibitor abiraterone and antiandrogens enzalutamide, apalutamide, and darolutamide. Both abiraterone and enzalutamide received initial FDA-approval based on modest survival benefit vs. placebo in men with mCRPC post-chemotherapy and subsequently in men treated pre-chemotherapy based on the results of the COU-AA-302 (abiraterone) and PREVAIL (enzalutamide) studies, Table 1.^7-10^ The effectiveness of first-line combination androgen blockade was revisited with multiple trials such as LATITUDE (abiraterone), STAMPEDE (abiraterone), ARCHES (enzalutamide), and TITAN (apalutamide) each demonstrating a significant survival advantage for combination therapy compared to ADT alone in men with high risk and metastatic PCa.^11-14^ Recent results from the ARASENS trial also demonstrated a survival advantage for “triplet” therapy consisting of ADT + docetaxel + darolutamide vs. the “doublet” of ADT + docetaxel.^15^ While these trials have shown significant improvement in survival vs. the control arm, once patients develop radiographic progression, survival is relatively short with median duration of survival in the range of 18-24 months.^11,12^

**Table 1.** Summary of clinical response to BAT in completed BAT studies in men with CRPC.

	*n*	PSA-PFS m	crPFS m	PSA50	OR	OS m
PILOT	14	NA	NA	50%	50% (5/10)	NA
RESTORE						
Post-enzalutamide (cohort A)	30	3.3	6.5	30%	50% (6/12)	NA
Post-abiraterone (cohort B)	29	NA	4.3	17%	29% (2/7)	NA
Post-ADT only (cohort C)	29	1	8.5	14%	31% (31%)	NA
TRANSFORMER						
BAT arm	94	2.8	5.7	28.2%	24.2% (8/33)	32.9
Enzalutamide arm	101	3.8	5.7	25.5%	4.2% (1/24)	29.0
*P*		.018	.4			.801
COMBAT (BAT + Nivolumab)	45	NA	5.7	40.0%	23.8% (10/42)	NA

Additionally, emerging data documents a significant reduction in response rate and response duration with sequential use of abiraterone or enzalutamide as “second- or third-line” ARSI therapy. Results from a series of small studies evaluating the use of enzalutamide after progression on abiraterone show a marked decrease in PSA PFS, time to progression, and objective response.^16-19^ Broadly, ~95% of patients respond to initial androgen ablative therapy, with ~65% response to first-line ARSI and ~25% response to second and further lines of ARSI therapy. Evaluation of patient biopsies and autopsy studies demonstrates that CPRC cells resistant to ARSI continue to engage in AR signaling and adapt to chronic exposure to low testosterone through a progressive, auto-regulatory increase in AR expression to sufficient levels to restore AR axis activity even in the absence of ligand.^20-22^ At each stage, resistance first manifests as a sustained rise in the androgen-responsive gene PSA, consistent with the reactivation of a functioning AR axis.

### Rationale for Supraphysiologic Testosterone as Therapy for CRPC

**Figure 1.**
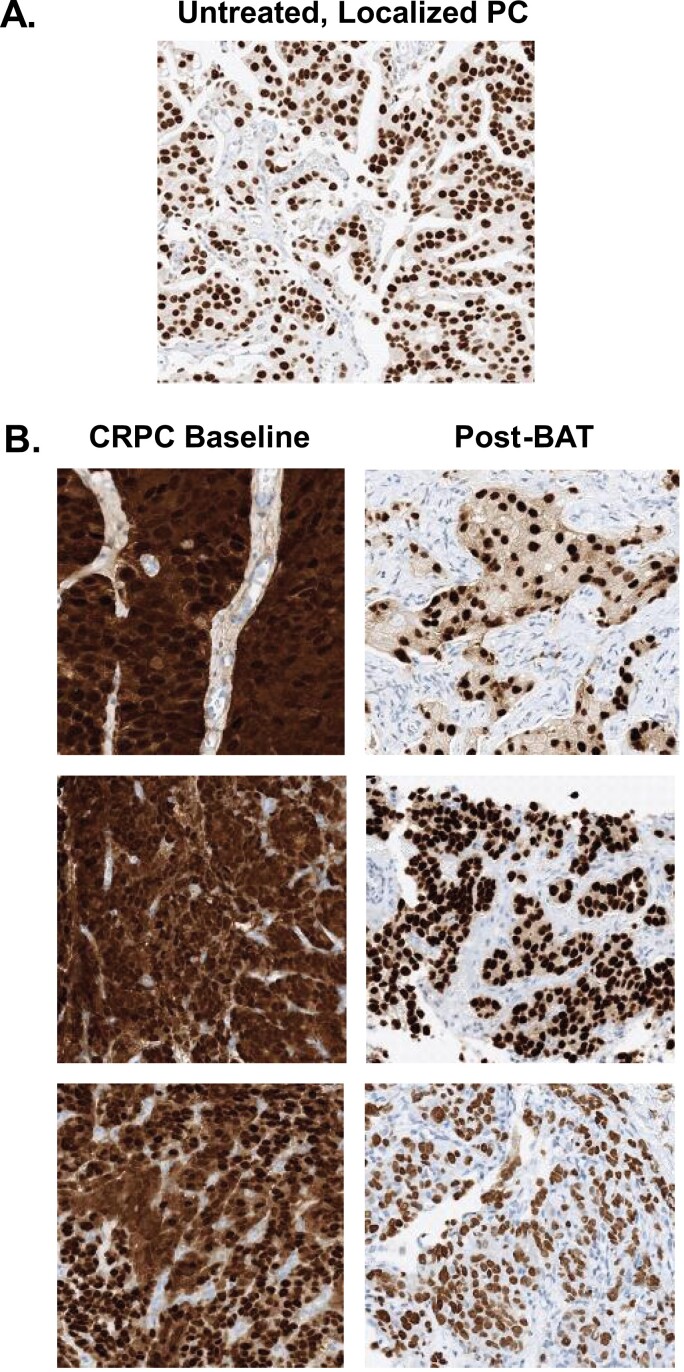
(**A**) AR expression in untreated, localized, castration sensitive PCa. (**B**) Representative examples of AR expression in lymph node biopsies from 3 patients at baseline and after 3 cycles of BAT.

A major factor driving resistance to ADT is the ability of PCa cells to adapt to chronic low androgen conditions by upregulating AR activity through overexpression, gene amplification, and expression of transcriptionally active AR variants that lack the ligand-binding domain, Fig. 1A, 1B.^23-27^ Data from a variety of studies has demonstrated that AR expression persists even in men with CRPC who have died from PCa despite having received chronic ADT and multiple types of ARSI.^20,25^ Chen et al. demonstrated that PCa cell lines adapt to serial passage in castrated mice through an auto-regulatory increase in AR expression that is sufficient to induce resistance to both ADT and the anti-androgen bicalutamide.^28^ Isaacs et al. documented that AR levels increased 30-90 fold in CRPC cell lines and clinical samples compared to normal prostate cells.^29^ While this marked upregulation of AR can drive resistance to ADT, it also creates a therapeutic vulnerability to treatment with supraphysiologic androgen (SPA).^28-30^ SPA can lead to growth arrest or cell death in CRPC cell models. This paradoxical effect initially was extensively demonstrated by the work of Dr Shutsung Liao, whose laboratory showed that PCA cells adapted to grow in low androgen conditions had high AR levels and were growth-inhibited by SPA in vivo.^31^ Over time, these xenografts became resistant to SPA through downregulation of the AR, making them once again sensitive to ADT.

Several complementary mechanisms for this paradoxical effect of SPA have been described. Isaacs et al. demonstrated that AR is a DNA licensing factor that plays a critical role in DNA replication and must be degraded as the cell goes through cycle.^21,22,29^ AR over-stabilization by SPA inhibits DNA re-licensing resulting in death in the subsequent cell cycle.^21,22^ Haffner et al. showed that SPA generates transient double strand DNA breaks (DSBs) in CRPC cells through the recruitment of AR and topoisomerase II beta (TOP2B) to androgen response elements (AREs).^32^ SPA downregulates oncogenes and upregulates tumor suppressor expression, inhibits expression of the ligand-independent AR variant, AR-V7and produces profound metabolic and gene expression changes in CRPC cells that can lead to activation of autophagy and ferroptosis.^33-35^

Based on this extensive preclinical data, we instituted a clinical program to demonstrate the safety and efficacy of SPA, a treatment we have termed Bipolar Androgen Therapy (BAT), in men with metastatic CRPC. To date we have completed 4 studies: (1) a proof-of-concept pilot (*n* = 14), (2) RESTORE—testing efficacy and safety of BAT in men with CRPC progressing on ADT, abiraterone or enzalutamide (*n* = 88), (3) TRANSFORMER—a randomized study comparing BAT to enzalutamide in men progressing on abiraterone (*n* = 195), and (4) COMBAT—evaluating sequential BAT and nivolumab (*n* = 44).^36-40^

### What is Bipolar Androgen Therapy (BAT)?

Preclinical in vitro and in vivo studies using human CRPC models suggest that a dosing regimen that produces supraphysiologic testosterone (ie, SPA) levels is critical for achieving the desired pharmacologic effect on the target CRPC cells.^33^ Thus, BAT involves the administration of sufficient testosterone to achieve a supraphysiologic serum level in men with CRPC who are progressing on chronic ADT ± ARSI. The term “bipolar” is used because BAT involves rapid cycling between 2 polar extremes: from supraphysiologic back to near-castrate serum testosterone levels over a treatment cycle achieved through the administration of 400 mg of testosterone cypionate, a generic, FDA-approved depot form of testosterone that is injected intramuscularly into the buttocks.^41^ Testosterone can also be delivered via transdermal, transbuccal, intranasal, or oral preparations. However, achieving supraphysiologic levels of testosterone with these preparations would be cost-prohibitive and likely require non-FDA-approved dosing.

### Assessing Response to BAT in Clinical Studies

**Figure 2.**
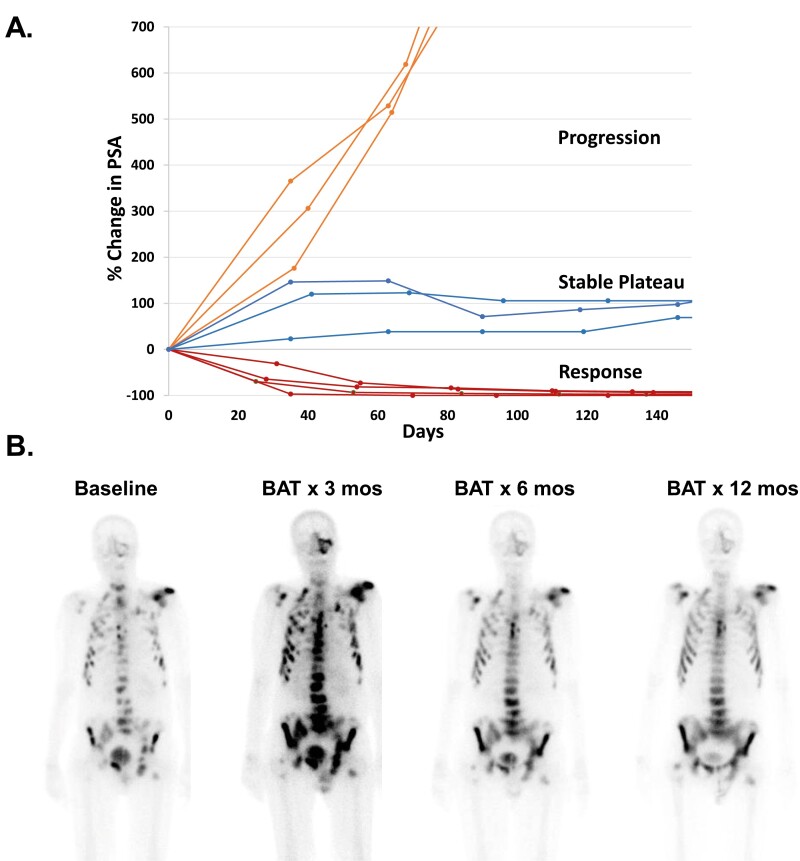
(**A**) Patterns of PSA response to BAT. Patients with PSA “Response” often show concurrent objective response. Patients with PSA “Stable Plateau” often have stable disease on scans. Patients with PSA “Progression” typically have concurrent radiographic progression. (**B**) Example of bone scan flare and resolution in a patient receiving BAT over 12 months.

The primary modalities used to assess therapeutic response in PCa are the serum PSA level, the CT scan, the bone scan, and clinical symptoms. PSA is an androgen-regulated gene whose expression is highly stimulated by testosterone and markedly inhibited by AR inhibitors, even in growth-arrested but viable PCa cells. Thus, reliance on PSA response alone may underestimate the effectiveness of BAT and, inversely, may overestimate the response to ARSIs. Three patterns of PSA response may be seen with BAT, Fig. 2A. A subset of patients have an initial increase in PSA after the first dose of BAT followed by a “Stable Plateau” phase. Patients with this PSA pattern have either no further increase or a slow increase in PSA. Those who have stable scans and are experiencing clinical benefits are often continued on BAT until radiographic progression is observed. For this reason, clinical and radiographic responses have been used as the primary endpoints in more recent BAT clinical studies.

In addition, BAT can also induce an initial “flare response” on the bone scan, Fig. 2B. This flare may be due to testosterone-stimulated release of inflammatory cytokines by PCa cells in the bone microenvironment leading to increased uptake of Tc-99m-methylene diphosphonate (MDP) on the bone scan at sites of metastatic disease. Therefore, careful attention must be paid to closely following the recommendation of the PCa Working Group 3 (PCWG3) in designing clinical trials and analyzing the initial response to BAT in the bone.^42^ This flare response is the basis of the ongoing BAT-RAD clinical trial (NCT04704505) designed to assess whether BAT can enhance the efficacy of Radium223 (Xofigo) in men with bone-predominant metastatic CRPC. BAT effects on PSMA-based PET imaging must also be considered, as PSMA-expression can be negatively regulated by androgens.^43,44^

## Clinical Studies of BAT in Men with CRPC

### Pilot Study of BAT

Based on extensive preclinical data supporting BAT, a pilot study was performed in which 16 asymptomatic CRPC patients with low to moderate metastatic burden were treated with testosterone cypionate (400 mg intramuscular; once every 28 days) and etoposide (100 mg oral daily; days 1-14 of 28).^34^ The rational for etoposide was based on findings that BAT could induce DSBs that could be stabilized by the TOP2B inhibitor etoposide.^31^ After 3 cycles, those with a declining PSA continued on BAT alone. BAT was well-tolerated and resulted in high rates of PSA50 (7/14) and objective responses (OR) (5/10 evaluable patients), Table 1. Although all men showed eventual PSA progression, 4 remained on BAT for ≥1 year. PSA50 response was observed in 90% (9/10) upon antiandrogen rechallenge post-BAT, consistent with restored sensitivity. This safety and efficacy in this initial proof-of-concept study supported further testing of BAT in larger clinical trials.

### Results From the RESTORE Study

The RESTORE study (NCT02090114) was an NIH-sponsored phase II study designed to assess BAT in men (*n* = 30/cohort) progressing on either enzalutamide (Cohort-A) or abiraterone (Cohort-B) or ADT alone (Cohort-C).^36,38^ The primary endpoint of the first part of the study was to assess PSA response to BAT. The second part was to determine in BAT could re-sensitize to repeat exposure to the ARSI that patients were progressing on prior to receiving BAT. Thus, patients progressing on enzalutamide were re-exposed to enzalutamide after BAT and similarly to abiraterone. PSA50 response to BAT was 30% in post-enzalutamide, 18% post-abiraterone, and 14% post-ADT alone, Table 1.^36-38^ PSA50 response in Cohorts-A and B was not significantly different in patients who had received 1 vs. 2 prior ARSI (ie, enzalutamide-abiraterone or abiraterone-enzalutamide). Adverse events (AEs) to BAT were primarily grades 1-2, with the most common being generalized musculoskeletal pain and sexual side effects that included breast tenderness, hot flashes, and gynecomastia. Serious AEs occurred in individual patients and were not attributed to BAT, with the exception of grade-3 hypertension in 3 patients.

### Results From the TRANSFORMER Study

**Figure 3.**
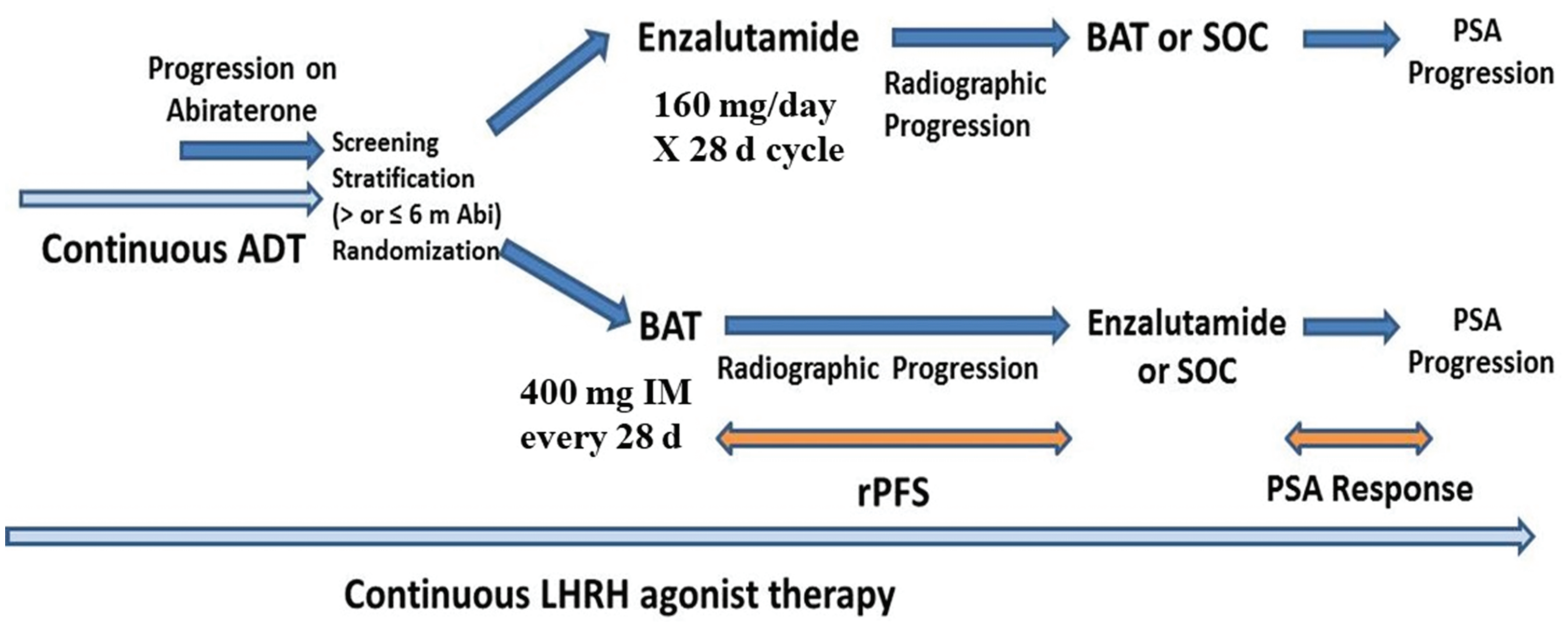
Trial design of the TRANSFORMER study.

The TRANSFORMER study (NCT02286921) was a DOD-sponsored randomized phase II study designed to compare the efficacy of BAT vs. enzalutamide in asymptomatic men with CRPC progressing on abiraterone, Fig. 3.^39^ The study was conducted in 195 patients who underwent 1:1 randomization to either standard dose enzalutamide (*n* = 101) or BAT (*n* = 94) at 400 mg IM every 28 days. The primary endpoint was clinical/radiographic progression free survival (crPFS). At the time of progression, patients were given the option to cross over to the alternate therapy. The trial did not meet the primary endpoint of improvement in crPFS relative to enzalutamide, with crPFS of 5.7 months in both arms (*P* = .2267), Table 1. OR was 24.4% for BAT and 4.2% for enzalutamide (*P* = .067). The best PSA50 response was 28.4% for BAT and 25.5% for enzalutamide (*P* = .697), Table 1. Overall, BAT was well tolerated with grades 1-2 and grades 3-5 fatigue compared to enzalutamide. BAT-treated patients experienced more generalized musculoskeletal pain and edema but less constitutional symptoms (eg, nausea, anorexia, depression, and insomnia) compared to enzalutamide.

**Figure 4.**
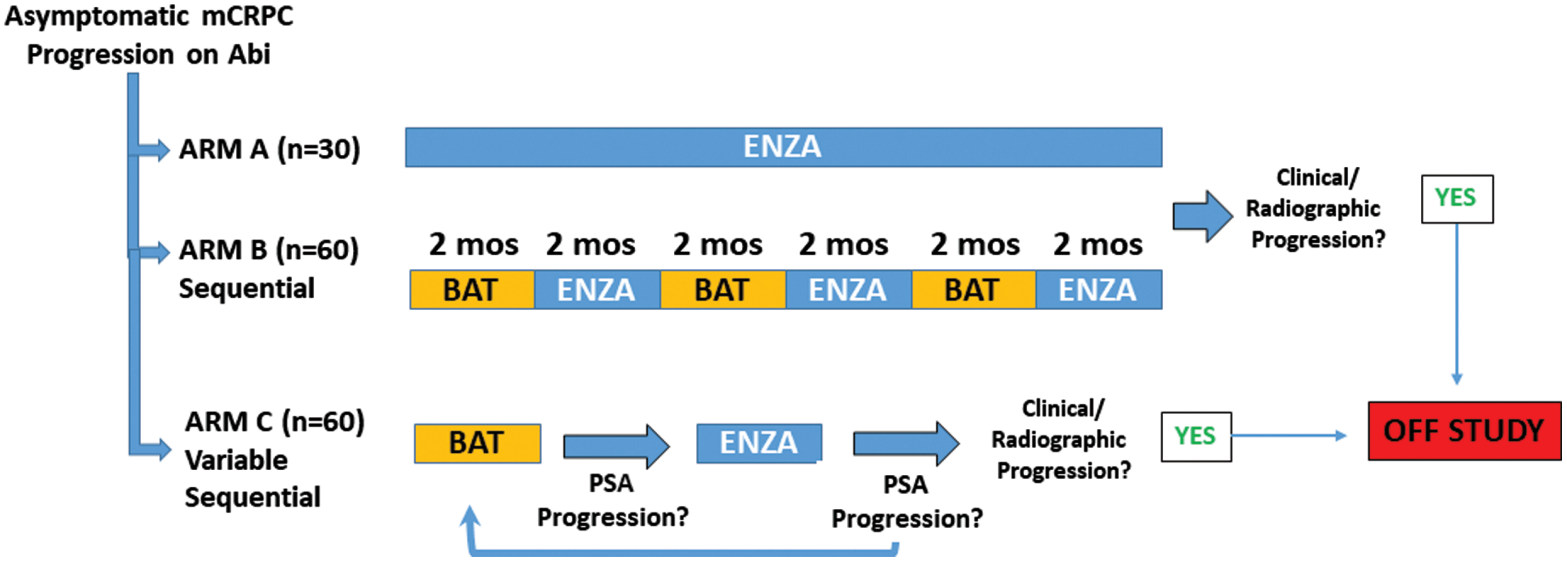
Trial design of the STEP-UP study.

### The COMBAT Trial

Based on preclinical data showing that SPA could activate interferon response pathways, and clinical data showing extreme response to anti-PD1 therapy in 3 men previously treated with BAT, we conducted a trial testing the efficacy of BAT followed by BAT plus Nivolumab (COMBAT study), (NCT03554317).^35,40,45^ This trial enrolled 44 patients with soft tissue lesions and required tumor biopsies at baseline and post-3 cycles of BAT, prior to initiation of Nivolumab. Prior treatment with multiple lines of ARSI and up to one prior taxane was allowed. The PSA50 response to BAT in these heavily pretreated patients was 40% and the OR was 24%, Table 1. The median rPFS was estimated at 5.7 months (95% CI, 4.9-7.8 months). Baseline biopsies in these 44 patients consistently showed that samples were poorly differentiated adenocarcinomas exhibiting universally high AR expression in both the cytoplasm and nucleus, Fig. 1B.^46^ After 3 cycles of BAT, total AR mRNA and protein levels declined, and AR protein was observed primarily in the nucleus, Fig. 1B.

### BAT Can Restore Sensitivity/Overcome Resistance to Antiandrogen Therapy

Prolonged exposure to SPA leads to a rapid adaptive downregulation of AR, which should have the potential to restore sensitivity to AR blockade. We first observed this potential re-sensitization in the initial pilot study of BAT where the PSA50 response to ARSI re-exposure was 90%, Table 2. To test this idea more intentionally, we designed the RESTORE study to determine whether BAT could re-sensitize patients to repeat exposure to the ARSI given prior to receiving BAT.^32,33^ Thus, patients progressing on enzalutamide were re-exposed to enzalutamide after BAT, and similarly to abiraterone. The PSA50 response to enzalutamide re-exposure was 71%, but only 21% for re-abiraterone exposure, Table 2. The reason for this differential response is not clear. One hypothesis is that levels of testosterone in prostate cancer tissue may remain elevated for some time after cessation of BAT. The effects of this residual testosterone on AR activity can be clocked by an antiandrogen such as enzalutamide, but not by testosterone-synthesis inhibition by abiraterone. The median time on enzalutamide pre-BAT was 8 months, while the median time to PSA-progression to enzalutamide rechallenge post-BAT was ~6 months. The RESTORE study included a third cohort (Cohort-C) progressing on ADT alone.^38^ After BAT, these patients were treated with first-line abiraterone or enzalutamide. In this group of 24 patients, nine with M_0_ and 15 with M_1_ CRPC, the PSA50 was 94% with a PSA90 response of 83%, Table 2.^38^ Remarkably, PSA-PFS in this group was 28.7 months, although this was a mix of M_0_ and M_1_ CRPC patients.

**Table 2.** Summary of clinical response to ARSI after BAT treatment.

		PSA-PFS m	crPFS m	PSA50 (%)	PSA90	PFS2 m	OS m
PILOT—any antiandrogen post-BAT	10	NA	NA	90	30%	NA	NA
RESTORE							
Enzalutamide post-BAT (cohort A)	22	5.5	4.7	68	9%	12.8	NA
Abiraterone post-BAT (cohort B)	19	NA	4.0	16	0%	8.1	NA
Enza/abi post-BAT (cohort C)	18	28.7	NR	94	83%	NR	NA
TRANSFORMER							
Enzalutamide post-BAT	36	10.9	NA	77.8	39	28.2	37.1
BAT post-enzalutamide	47	1.1	NA	21.3	2	19.6	30.2
*P*=		.0001				.015	.225
Enzalutamide post-abiraterone	35	NA	4.9	28.6	Ref.^15^		
	39	NA	2.8	12.8	Ref.^16^		
	102	3	NA	29	Ref.^17^		
	214	5.7	8.1	27	Ref.^18^		

In the TRANSFORMER study, at the time of progression 47 (49%) patients on enzalutamide crossed-over to BAT and 36 (33.7%) crossed from BAT to enzalutamide. Remarkably, the PSA50 response for patients who received enzalutamide after BAT was 77.8% compared to 25.5% for patients who received enzalutamide immediately after abiraterone. OR in the enzalutamide post-BAT patients was 28.6% compared to only 4.2% for enzalutamide given immediately post-abiraterone. Time to PSA progression was also markedly improved increasing almost 3-fold from 3.8 m post-abiraterone to 10.9 months post-BAT. Finally, post-hoc analysis of PSA progression to the first and second stages of the study (PSA-PFS2) for all patients revealed a median of 19.6 months for the sequence of enzalutamide crossing over to BAT but 28.2 months for a sequence of BAT crossing over to enzalutamide. In comparison, the PFS for patients receiving enzalutamide after abiraterone in this trial was 5.7 months, which is similar to PFS observed in other studies evaluating enzalutamide after abiraterone.^16,19^ Overall survival for patients receiving BAT and then enzalutamide was 37.1 months, compared to only 28.6 months for those receiving enzalutamide alone (HR 0.52, *P* = .031).

Taken together, these combined results support the conclusion that BAT can re-sensitize PCa cells to subsequent antiandrogen therapy with results that are superior to those observed in the multiple small studies assessing the efficacy of enzalutamide post-abiraterone, Table 2.^16-19^

### Predictors of Response to BAT

BAT produces PSA and objective responses in ~30% of patients with CRPC. Predictors and mechanisms of response and resistance are currently under study. Previously, Chatterjee et al., demonstrated that SPA could repress genes involved in DNA repair and delay the restoration of damaged DNA that was augmented by PARP1 inhibition.^30^ SPA-induced DSBs were accentuated in BRCA2-deficient PCas, and combining SPA with PARP or DNA-PKcs inhibition further repressed growth. This observation formed the basis of a clinical trial testing BAT + olaparib (NCT03516812) in men with CRPC.^47^ Next-generation sequencing performed on biopsies from PCa patients receiving BAT revealed that patients with mutations in genes mediating homology-directed DNA repair were more likely to exhibit clinical responses to BAT. Sena et al. recently demonstrated that growth inhibition of PCa models by SPA required high AR activity and was driven in part by downregulation of MYC.^46^ Using matched sequential patient biopsies, AR activity scores were generated based on expression of 10 canonical AR-target genes. High AR activity in pretreatment biopsies predicted downregulation of MYC, clinical response, and prolonged progression-free and overall survival for patients on BAT. BAT induced strong downregulation of AR in all patients, which was shown to be a primary mechanism of acquired resistance to SPA. Acquired resistance could be overcome by alternating SPA with the enzalutamide, which induced adaptive upregulation of AR and re-sensitized PCa to SPA. This work identified high AR activity as a predictive biomarker of response to BAT and supported a treatment paradigm for PCa involving repeat cycling between AR inhibition and activation.^48^ This concept is being tested in the ongoing STEP-UP trial (NCT04363164), which involves repetitive sequencing between BAT and enzalutamide using two different sequencing schedules.

### BAT Can Be Given Safely to Men With Asymptomatic CRPC

At the outset, the major concern for use of BAT in men with mCRPC was the potential to stimulate PCa cell proliferation leading to rapid progression and inducing worsening symptoms particularly worsening bone pain. Earlier studies assessing testosterone in men with symptomatic PCa demonstrated the potential for significant worsening of pain that could occur often within hours to days of treatment with testosterone. On this basis, across all BAT studies we have limited eligibility to patients who are asymptomatic with no bone pain due to PCa and no worrisome lesions (ie, pending spinal cord compression, and bone fracture) that could cause severe symptoms in the event of tumor progression.^34,36-40^ We also excluded patients with urinary obstruction requiring catheterization due to enlarged prostate secondary to PCa or benign prostatic hypertrophy (BPH).

With those caveats, to date we have observed that BAT is remarkably safe in men with asymptomatic mCRPC. In the RESTORE study, adverse events to BAT were primarily grades 1-2 with the most common being generalized musculoskeletal pain and sexual side effects that included breast tenderness, hot flashes, and gynecomastia.^36,37^ Serious adverse events occurred in individual patients and were not attributed to BAT with the exception of grade 3 hypertension that occurred in 3 patients.

In the TRANSFORMER study comparing BAT to enzalutamide, the majority of AEs were grade 1-2; serious AEs were comparable in the 2 arms and occurred in 19.1% of patients on BAT and 20.6% on enzalutamide.^39^ Only one grade 5 AE (death not otherwise specified) was observed in a patient on enzalutamide. Grade ≥3 AEs were primarily due to worsening generalized, back or extremity pain (*n* = 7, 7.9%) in the BAT group and pain (*n* = 10, 10.35), fatigue (*n* = 7, 7.2%), and hypertension (*n* = 4, 4.1%) in the enzalutamide group. The incidence of AEs was generally similar in the 2 groups. Notable exceptions included fatigue with 48.5% of patients on enzalutamide experiencing grades 1-2 and 7.2% grade 3-4 fatigue, compared with 31.5% of BAT patients experiencing only grade 1-2 fatigue. Enzalutamide was associated with a higher percentage of grades 1-2 constitutional symptoms such as anorexia, depression, anxiety, insomnia, headache, and generalized muscle weakness as well as GI complaints (diarrhea, constipation, abdominal pain, and flatulence). BAT was associated with grades 1-2 increased endocrine side-effects (hot flashes, breast tenderness, and gynecomastia) and musculoskeletal complaints (peripheral edema and generalized musculoskeletal pain). Most patients on BAT experienced a significant increase in hemoglobin levels that returned to baseline once BAT was discontinued.

### BAT Improves Quality of Life in Men With CRPC

In the initial pilot study, many patients reported improvement in overall quality of life (QoL) and sexual function. To formally evaluate this effect, in the RESTORE study patients completed QoL questionnaires using validated instruments.^36^ Significant improvement was observed on SF-36 Instrument subscales of Physical Function, Emotional Well-Being, and Energy-Fatigue, on the FACIT Fatigue Scale and on the International Index of Erectile Function (IIEF) survey for patients receiving BAT compared to baseline. No significant difference was seen in the PANAS-SF screens for positive or negative response. The TRANSFORMER study evaluated QoL for patients on BAT vs. Enzalutamide. Patients on BAT showed significant improvement compared to enzalutamide on the same SF-36 Instrument subscales, on the FACIT Fatigue Scale, and on the IIEF survey.^39^ In a recent study designed to assess the effect of BAT on body composition, image analysis of computed tomography imaging at baseline and after 3 cycles of BAT was performed on 60 patients from the RESTORE and TRANSFORMER trials.^49^ Cross-sectional areas of psoas muscle, visceral, and subcutaneous fat were measured at the L3 vertebral level. Overall, patients lost a mean of 7.8% of subcutaneous fat, 9.8% of visceral fat, and gained 12.2% muscle mass. However, change in body composition did not correlate with improvements in the FACIT-Fatigue or SF-36 subscales.^49^

### Conclusions/Recommendations

Our cumulative clinical experience over the past 10 years treating >250 CRPC patients establishes the meaningful clinical activity and safety of BAT and supports additional studies to determine its optimal clinical integration. Key findings from these clinical studies are that BAT (a) can be safely administered to asymptomatic patients with mCRPC; (b) does not produce symptomatic disease progression; (c) produces sustained PSA and objective responses in 30%-40% of patients; and (d) can re-sensitize and prolong response to subsequent antiandrogen therapy.^34,36-40^ While ADT for advanced PCa often produces debilitating sexual and metabolic side effects, another highly significant feature of this approach is that BAT can make men feel remarkably better by decreasing fatigue, increasing physical activity, and restoring libido and sexual function. BAT can also increase skeletal muscle tone and decrease visceral and subcutaneous fat.^48^ Thus, the incorporation of inexpensive, high-dose testosterone via BAT into the treatment paradigm for men with CRPC has the potential to improve quality of life (QoL) and minimize morbidity from the metabolic sequelae produced by androgen ablative therapies. Patients who are interested in more information on BAT can be referred to a recent review written for a lay audience.^48^

Ongoing clinical trials are designed to assess the optimal way to sequence and combine BAT in PCa. Patients are encouraged to seek out and participate in such clinical trials when possible. For those without access to trials, there are 2 clinical settings in which BAT could be recommended based on the available clinical data. First, for asymptomatic patients who have initially progressed on ADT, BAT could be administered prior to starting an ARSI due to its marked ability to enhance subsequent response to these agents. Second, in men with CRPC progressing on abiraterone, BAT can be considered as part of sequential therapy with an antiandrogen based on results from the TRANSFORMER study. Importantly, BAT should always be given in conjunction with ongoing ADT. BAT is not to be used in patients with castration-sensitive disease outside of a clinical trial. BAT is also not currently recommended for use in patients with PCa bone pain as these patients are at risk for significant worsening of pain following testosterone injection. In those few patients who experienced pain flares in BAT clinical studies, we have observed that the flare usually occurs within 12-48 h post-testosterone injection. Pain can be treated with anti-inflammatory medications but may be severe enough to require narcotics. It typically resolves after ~1-week post-BAT, when the testosterone level begins to fall. For those patients who do develop a pain flare on the first dose of BAT that resolves by the end of a 28-day cycle, consideration can be given to continue BAT for a second cycle. We have observed pain improvement or resolution that did not return with subsequent cycles of BAT. For those patients who continue to have pain at the end of the first 28-day cycle, BAT should be discontinued.

### Future Directions

Although our initial results have been encouraging, with some men responding to BAT for several years, the median duration of progression-free survival across these studies is approximately 6-9 months. Thus, rational combinatorial approaches are needed that can further enhance and prolong the efficacy of BAT. Combinations based on the current understanding of the profound effects of androgen on PCa cell gene expression, metabolism, and immune microenvironment are being explored in preclinical and clinical studies. These include BAT in combination with Nivolumab (NCT03554317),^40^ olaparib (NCT03516812), ^223^Radium (NCT04704505), and carboplatin (NCT03522064). Finally, although BAT has activity as a single agent, more importantly the sequence of BAT→ARSI (such as enzalutamide) should be considered as a therapeutic continuum. On this basis, the eficacy of repeat cycling between BAT and enzalutamide is being tested in the Sequential Testosterone and Enzalutamide Prevents Unfavorable Progress (STEP-UP) trial, Fig. 4. Further studies are required to assess the disease stage and optimal timing for sequencing between BAT and ARSI based on an understanding of PCa cells adaptive response to changing AR activity in the tumor microenvironment.

## Data Availability

No new data were generated or analyzed in support of this research.
